# Updating contextualized clinical practice guidelines on stroke rehabilitation and low back pain management using a novel assessment framework that standardizes decisions

**DOI:** 10.1186/s13104-015-1588-8

**Published:** 2015-11-04

**Authors:** Ephraim D. V. Gambito, Consuelo B. Gonzalez-Suarez, Karen A. Grimmer, Carolina M. Valdecañas, Janine Margarita R. Dizon, Ma. Eulalia J. Beredo, Marcelle Theresa G. Zamora

**Affiliations:** Department of Physical Medicine and Rehabilitation, University of Santo Tomas Hospital, Manila, Philippines; International Center for Allied Health Evidence (iCAHE), University of South Australia, Adelaide, Australia; Department of Rehabilitation Medicine, St. Luke’s Medical Center, Quezon City, Philippines; College of Rehabilitation Sciences, University of Santo Tomas, Manila, Philippines; Department of Rehabilitation Medicine, Philippine Orthopedic Center, Quezon City, Philippines; Philippine Academy of Rehabilitation Medicine (PARM), Quezon City, Philippines

**Keywords:** Clinical practice guidelines, Philippines, Updating guidelines, Evidence-based practice, PARM

## Abstract

**Background:**

Clinical practice guidelines need to be regularly updated with current literature in order to remain relevant. This paper reports on the approach taken by the Philippine Academy of Rehabilitation Medicine (PARM). This dovetails with its writing guide, which underpinned its foundational work in contextualizing guidelines for stroke and low back pain (LBP) in 2011.

**Methods:**

Working groups of Filipino rehabilitation physicians and allied health practitioners met to reconsider and modify, where indicated, the ‘typical’ Filipino patient care pathways established in the foundation guidelines. New clinical guidelines on stroke and low back pain which had been published internationally in the last 3 years were identified using a search of electronic databases. The methodological quality of each guideline was assessed using the iCAHE Guideline Quality Checklist, and only those guidelines which provided full text references, evidence hierarchy and quality appraisal of the included literature, were included in the PARM update. Each of the PARM-endorsed recommendations was then reviewed, in light of new literature presented in the included clinical guidelines. A novel standard updating approach was developed based on the criteria reported by Johnston et al. (Int J Technol Assess Health Care 19(4):646–655, 2003) and then modified to incorporate wording from the foundational PARM writing guide. The new updating tool was debated, pilot-tested and agreed upon by the PARM working groups, before being applied to the guideline updating process.

**Results:**

Ten new guidelines on stroke and eleven for low back pain were identified. Guideline quality scores were moderate to good, however not all guidelines comprehensively linked the evidence body underpinning recommendations with the literature. Consequently only five stroke and four low back pain guidelines were included. The modified PARM updating guide was applied by all working groups to ensure standardization of the wording of updated recommendations and the underpinning evidence bases.

**Conclusions:**

The updating tool provides a simple, standard and novel approach that incorporates evidence hierarchy and quality, and wordings of recommendations. It could be used efficiently by other guideline updaters particularly in developing countries, where resources for guideline development and updates are limited. When many people are involved in guideline writing, there is always the possibility of ‘slippage’ in use of wording and interpretation of evidence. The PARM updating tool provides a mechanism for maintaining a standard process for guideline updating processes that can be followed by clinicians with basic training in evidence-based practice principles.

**Electronic supplementary material:**

The online version of this article (doi:10.1186/s13104-015-1588-8) contains supplementary material, which is available to authorized users.

## Background

The PARM group first published two Filipino-contextualized clinical practice guidelines (CPGs) on stroke rehabilitation, and low back pain, in 2012. They are freely available on the PARM website [1, 2], and have since been the subject of nation-wide baseline audit and implementation activity [3–5]. The PARM clinical practice guidelines for stroke and low back pain have been endorsed by the International Society of Physical and Rehabilitation Medicine (ISPRM) which provided the members’ recommendations for best practice in the field of rehabilitation medicine. The low back pain guideline has been submitted to the Philippine Health Insurance Corporation (PHIC), and now serves as the basis for reimbursement of fees for the management of low back pain which includes rehabilitation consultation, physical therapy treatments, non-surgical interventions such as acupuncture and epidural steroid injections; and diagnostic procedures such as spine X-ray, magnetic resonance imaging and electromyography. This will be ground-breaking in a developing country such as the Philippines because rehabilitation services are presently not being subsidized by the government health insurance agency.

In line with international recommendations regarding the importance of the currency of evidence in clinical guidelines [6, 7], the 2012 Filipino-contextualized stroke and low back pain guidelines were due for revision and updating in 2014. Updating guidelines is an essential process that incorporates the best new evidence in its recommendations, using relevant new scientific research including new technologies in the diagnostic and treatment alternatives, economic differences or changes in values and preferences [8]. The updating process consists of the identification of new evidence, the assessment whether the new evidence warrants an update, and the formulation of new or modified recommendations [8, 9].

However, there are only a few publications that reported methods and approaches for updating guidelines [10–13]. This appears to reflect the greater emphasis placed on methods for developing ‘de novo*’* (new) evidence based clinical practice guidelines [14]. International studies have consistently shown that common difficulties encountered in updating guidelines are: (1) the most appropriate timeframe within which guidelines should be updated; (2) lack of guidance in choosing a rigorous, efficient, standardized updating process; (3) the lack of efficient monitoring systems to identify new, potentially-relevant evidence; (4) decisions regarding extent of updating (whether updates should be partial or full); and (5) the cost effectiveness of updating a practice guideline [6, 7, 14].

Thus, as with guideline contextualization, the PARM group found itself without international guidance, and faced with this question: how do we update clinical practice guidelines using contextually-relevant principles? These principles need to provide a transparent, standard and comprehensive platform which all team members could use, when updating the current stroke and low back pain guidelines, and which could be used ongoing to update other contextualized guidelines as necessary.

This paper reports on the approach taken by PARM to develop a standard tool to assist clinicians involved in updating clinical guidelines in developing countries. This tool dovetails with the PARM writing guide [3], which underpinned the development of the foundation PARM contextualized guidelines for stroke and low back pain.

## Methods

Ethical approval was obtained from the ethics review board of the Philippine Academy of Rehabilitation Medicine. Informed consent was obtained from each member of the working group involved in this study.

As occurred for the original PARM guidelines, all updating work was voluntarily undertaken by a working party of approximately 35 physiatrists (rehabilitation doctors) who were members of the PARM. Participants were invited for their research background and willingness to contribute. The group ranged in age and clinical experience, and approximately 75 % had not been involved in the foundational contextualization process 3 years before. The group met for an intensive weekend of training, discussion and debate about updating processes, and to develop the updating tool. Then smaller working groups undertook to update specific sections of the stroke and low back pain guidelines. Each group applied the updating tool to one or more recommendations before the weekend workshop finished, so that any concerns regarding the updating process could be identified and clarified.

To assist in standardizing the guideline contextualization process used in the development of the original guidelines, a PARM writing guide was established [3]. This guide establishes a uniform framework for summarizing differently-worded recommendations and differently-reported strengths of the body of evidence for recommendations extracted from the included guidelines, relevant to a particular situation in the Filipino patient journey. The guide is to be used in the event that there are: more than one relevant recommendation extracted from the relevant guidelines, which addresses a particular aspect of the Filipino patient journey, and/or different methods of reporting the underpinning strength of the body of evidence of the relevant recommendations from the included guidelines. All relevant recommendations (to the patient journey) were collated in a table for each element of the journey, along with the underpinning levels of evidence, and the guideline reference from which the recommendation had been extracted. The concept of *uniform thought* was coined by the PARM group to identify similar intent, from differently worded recommendations from different guidelines. This was found to be a critical step in the contextualizing process, because guidelines formed the source of recommendations and the evidence base, rather than individual literature. This meant that the working parties often needed to resolve the issue of different wording in recommendations, despite the same intent of the recommendation and the same underpinning references. The evidence body was thus described in six different categories by the PARM contextualization process [3] (Table 1).

**Table 1:** The PARM writing guide (Gonzalez-Suarez et al. [3], page 150)

1. There is strong evidence	Consistent grades of high-quality evidence with uniform thought, and at least a moderate volume of references to support the recommendation(s)
2. There is evidence	A mix of moderate- and high-quality evidence with uniform thought and at least a low volume of references; OR A mix of high- and low-quality evidence with uniform thought and high volume of references; OR High-level evidence coupled with GPPs, and at least moderate volume of references; OR One level I paper with at least moderate volume of references
3. There is some evidence	Single level II (A) paper; OR Inconsistent grades of high and low evidence with uniform thought and moderate volume of references; OR Consistent grades of low-level evidence with uniform thought and at least a moderate volume of references
4. There is conflicting evidence	A mix of levels of evidence with non-uniform thought, irrespective of the volume of evidence
5. There is insufficient evidence	Low or inconsistent levels of evidence with low volume references with or without GPPs
6. There is no evidence	Absence of evidence for any aspect of the patient journey

These categories were then synthesized into the wording that PARM used to present the recommendations which had been distilled from the included guidelines during the 2012 contextualization process. These were PARM Strongly Endorses, PARM Endorses, PARM Recommends, PARM Suggests or PARM Does not endorse.

The new evidence body against which recommendations in the 2012 PARM-contextualized stroke and low back pain guidelines were assessed, consisted of primary and secondary literature published since 2011, and which were reported in the new clinical guidelines. The 2011 date was set, because the 2012 guidelines included in the contextualization process, were based on literature searches up until 2011.

Clinical guidelines in stroke rehabilitation and low back pain management published internationally since 2011 were sought from a comprehensive search of the following electronic databases: PubMed, Google Scholar, National Institute for Health and Clinical Excellence (NICE), Scottish Intercollegiate Guidelines Network (SIGN), National Health and Medical Research Center (NHMRC), New Zealand Guidelines Group (NZGG), National Guidelines Clearinghouse (NGC), using key words of clinical practice guidelines and stroke rehabilitation (or low back pain). Criteria for inclusion in the updating process were: that the guideline included rehabilitation recommendations; the guideline was available in full text; it was published in English language; published since 2010 (the close-off date of literature inclusion for the inaugural PARM guidelines); and not included in the original contextualized PARM guidelines. Moreover, only de novo guidelines, or comprehensive updates of guidelines (which included new literature) were included. Guidelines that re-stated, or adopted the findings of other guidelines, without adding to the body of literature, were excluded.

The 14-item guideline appraisal tool by the International Centre for Allied Health Evidence (iCAHE) was applied to each guideline [15]. Particular attention was paid to the items related to availability of information on the hierarchy of evidence and the quality of studies which underpinned recommendations. If information was inadequate or not available, the guideline could not be included in the updating process. To be included in the study, PARM determined that a score of 10 should be used as the quality cut-off (71 % quality criteria met).

The basis for PARM updating used the four levels proposed by Johnston and his colleagues [10]. The specifications of the PARM writing guide for strength of evidence base, uniformity of thought and volume of references were amalgamated with the Johnston et al. [10] guideline updating approach (Table 2). A number of different descriptions of the synthesized information were presented to the PARM writing group for consideration, and then the preferred options were trialled. Modifications were made based on the utility and reliability of the guide, when it was applied to different evidence examples, and a final version was agreed upon.

**Table 2:** Updating process (Johnston et al. [10], page 648)

Level 1	The new evidence is consistent with the data used to inform the original practice guideline report. The recommendations in the original report remain unchanged
Level 2	The new evidence is consistent with the data used to inform the original practice guideline report. The strength of the recommendations in the original report has been modified to reflect this additional evidence
Level 3	The new evidence is inconsistent with the data used to inform the original practice guideline report. However, the strength of the new evidence does not alter the conclusions of the original document. Recommendations in the original report remain unchanged
Level 4	The new evidence is inconsistent with the data used to inform the original practice guideline report. The strength of the new evidence will alter the conclusions of the original document. Recommendations in the original report will change

**Fig. 1:**
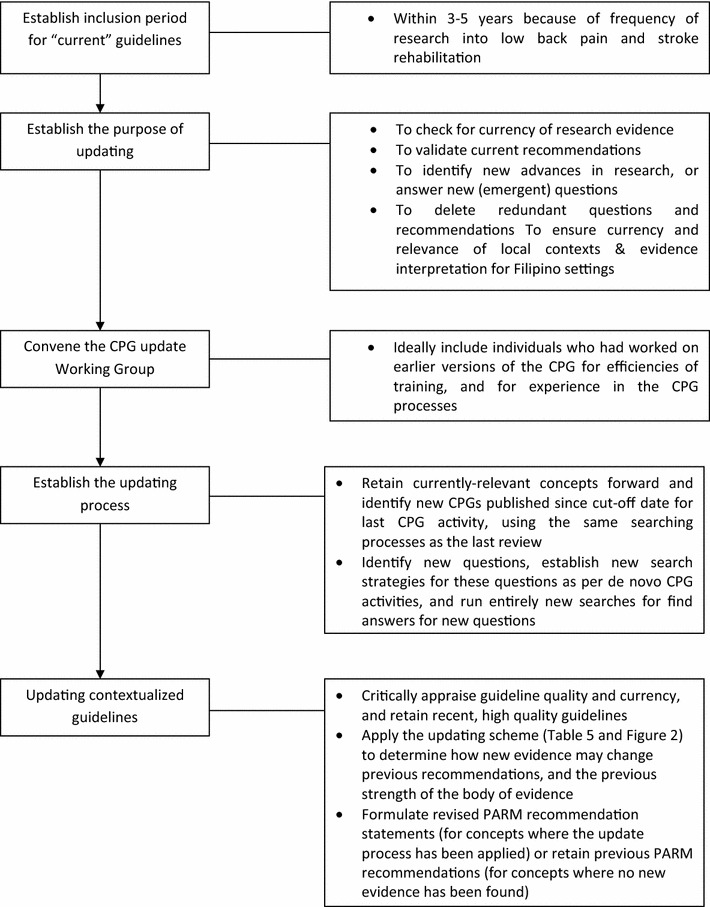
Flow diagram of process in updating guidelines

A flow diagram of process in updating contextualized guidelines is shown as Fig. 1.

## Results

Additional file 1: Appendix 1 describes the consort diagrams for the CPG inclusion process for stroke and low back pain. Ten new guidelines on stroke and eleven for low back pain were identified. For the management of low back pain, four of the eleven clinical practice guidelines initially identified did not meet the inclusion criteria: two were not readily available via the internet [16, 17]; one was not published in English [18] one was not a de novo or updated CPG [19]. For the stroke rehabilitation CPGS, of the ten clinical practice guidelines that were initially identified, three were excluded as there was no rehabilitation aspect included in the recommendations [20–22] while another was excluded because it did not rank the quality of the evidence and did not link the hierarchy of evidence to its recommendations [23]. Instead, it used a formal consensus approach in formulating the recommendations. The PARM group agreed that this would not make it possible to compare the quality of evidence with other guidelines.

Seven potentially relevant CPGs for low back pain were critically appraised and three CPGs scored less than 71 % [24–26]. Therefore only four CPGs were included in the revision of the PARM contextualized CPG for low back pain [27–30] (see Table 3). Six potentially-relevant CPGs for stroke were assessed for methodological quality, and one was excluded due to a low methodological score (9/14) [31]. The remaining five guidelines were included in the update [32–36] (see Table 4).

**Table 3:** Quality Scores of the Low Back Pain Clinical Practice Guidelines Using the International Centre for Allied Health Evidence (iCAHE) Guideline Quality Checklist [15]

	APTA 2012^a^ [27]	ICSI 2012^b^ [28]	OTTAWA 2012^c^ [29]	TOP 2011^d^ [30]	KNGF 2013^e^ [24]	GHC 2013^f^ [25]	RCC 2014^g^ [26]
1. Availability							
Is the guideline readily available in full text?	1	1	1	1	1	1	1
Does the guideline provide a complete reference list?	1	1	1	1	0	1	1
Does the guideline provide a summary of its recommendations?	1	1	1	1	1	0	1
2. Date							
Is there a date of completion available?	1	1	1	1	1	1	1
Does the guideline provide an anticipated review date?	1	1	0	0	0	0	1
Does the guideline provide dates for when literature was included?	1	1	1	1	0	1	1
3. Underlying evidence							
Does the guideline provide an outline of the strategy they used to find underlying evidence?	1	1	1	1	0	0	0
Does the guideline use a hierarchy to rank the quality of the underlying evidence?	1	1	1	1	0	0	0
Does the guideline appraise the quality of the evidence which underpins its recommendations?	1	1	1	1	0	0	0
Does the guideline link the hierarchy and quality of underlying evidence to each recommendation?	1	1	1	1	0	0	0
4. Guideline developers							
Are the developers of the guideline clearly stated?	1	1	1	1	1	0	0
Does the qualifications and expertise of the guideline developer(s) link with the purpose of the guideline and its end users?	1	1	1	1	1	1	0
5. Guideline purpose and users							
Are the purpose and target users of the guideline stated?	1	1	1	1	1	1	1
6. Ease of use							
Is the guideline readable and easy to navigate?	1	1	1	1	1	1	1
Total score	14	14	13	13	7	7	8

^a^ APTA 2012: [27]

^b^ ICSI 2012: [28]

^c^ OTTAWA 2012: [29]

^d^ TOP 2011: [30]

^e^ KNGF 2013: [24]

^f^ GHC 2013: [25]

^g^ RCC 2014: [26]

**Table 4:** Quality scores of the stroke rehabilitation clinical practice guidelines using the International Centre for Allied Health Evidence (iCAHE) Guideline Quality Checklist [15]

	AHA women 2014^a^ [34]	AHA stroke and TIA 2014^b^ [35]	AHA ischemic 2013^c^ [36]	NICE 2013^d^ [32]	NZGG 2010^e^ [33]	SASS 2010^f^ [31]
1. Availability						
Is the guideline readily available in full text?	1	1	1	1	1	1
Does the guideline provide a complete reference list?	1	1	1	1	1	1
Does the guideline provide a summary of its recommendations?	1	1	1	1	1	1
2. Date						
Is there a date of completion available?	1	1	1	1	1	1
Does the guideline provide an anticipated review date?	0	1	0	0	1	0
Does the guideline provide dates for when literature was included?	1	1	0	1	1	0
3. Underlying evidence						
Does the guideline provide an outline of the strategy they used to find underlying evidence?	1	1	1	1	1	0
Does the guideline use a hierarchy to rank the quality of the underlying evidence?	1	1	1	1	1	1
Does the guideline appraise the quality of the evidence which underpins its recommendations?	1	1	1	1	1	0
Does the guideline link the hierarchy and quality of underlying evidence to each recommendation?	1	1	1	1	1	0
4. Guideline developers						
Are the developers of the guideline clearly stated?	1	1	1	1	1	1
Does the qualifications and expertise of the guideline developer(s) link with the purpose of the guideline and its end users?	1	1	1	1	1	1
5. Guideline purpose and users						
Are the purpose and target users of the guideline stated?	1	1	1	1	1	1
6. Ease of use						
Is the guideline readable and easy to navigate?	1	1	1	1	1	1
Total score	13	14	12	13	14	9

^a^ AHA women 2014: [34]

^b^ AHA stroke and TIA 2014: [35]

^c^ AHA ischemic 2013: [36]

^d^ NICE 2013: [32]

^e^ NZGG 2010: [33]

^f^ SASS 2010: [31]

As described in Gonzalez-Suarez et al. [3], designing patient journey*s* and mapping the steps in them to CPG Recommendations were a critical part of the contextualization process. Thus they were revisited in the 2014 PARM workshop with the focus on assessing generalizability and applicability of the existing guideline recommendations for the two conditions of interest using NHMRC FORM [37] and PARM context points. This process ensured that all the essential steps from initial presentation to discharge from health care services were included, and that new members of the PARM working group were conversant with the patient journey process underpinning the contextualization process of guideline recommendations. This step involved much discussion on the elements of the patient journey, how the previous guidelines’ patient journeys could be improved upon in order to better embed comprehensive and clinically useful recommendations. An example of how the patient journey was modified between 2012 and 2014 is provided in Additional file 1: Appendix 2.

**Fig. 2:**
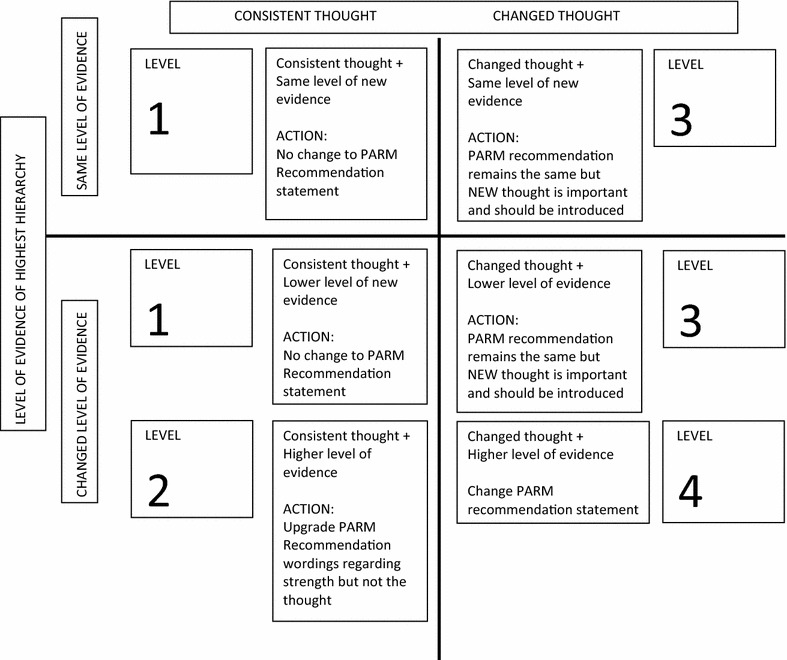
PARM writing guide in revising a recommendation

After much debate, the working group agreed that the PARM updating tool should be presented in two different ways (see Table 5; Fig. 2).

**Table 5:** PARM writing guide on updating a recommendation

Level	Johnston et al. [1] description	PARM description (old vs new comparison)	Action
I	The new evidence is consistent with the data used to inform the original practice guideline report. The recommendations in the original report remain unchanged	Consistent thought + same level of new evidence or consistent thought + lower level of new evidence	No change in PARM evidence rating
II	The new evidence is consistent with the data used to inform the original practice guideline report. The strength of the recommendations in the original report has been modified to reflect this additional evidence	Consistent thought + higher level of new evidence	Upgrade PARM evidence rating
III	The new evidence is inconsistent with the data used to inform the original practice guideline report. However, the strength of the new evidence does not alter the conclusions of the original document. Recommendations in the original report may remain unchanged but new information could be introduced	Inconsistent thought with same or lower level of new evidence	No change in evidence rating, but new evidence must be presented in the evidence summary column Requires new wording to inform practice and flags possible future changes (PARM recommendation may change in the future)
IV	The new evidence is inconsistent with the data used to inform the original practice guideline report. The strength of the new evidence will alter the conclusions of the original document. Recommendations in the original report will change. This change is a priority for the working party members. Modifications to the guideline are now in progress	Inconsistent thought with higher level new evidence	Change PARM recommendation

This guidance was found to be consistently valuable across patient journey points for stroke and low back pain, recommendations and evidence types and sources. This tool assisted the working groups to resolve any dilemma which arose when amalgamating new evidence with old. The most difficult classification was found to be Level 3, where the recommendation itself was not necessarily reworded, but additional statements were sometimes required to incorporate new information which had been reported in recent clinical guidelines. This often related to emergent new technology or refinement of effective treatment techniques (for instance exercise programs). Examples of updated recommendation statements are provided in Table 6.

**Table 6:** Format for the summary of recommendations with sample statements

2011 (old) recommendation statement	2011 (old) evidence grade	2011 (old) source guidelines	2014 (new) evidence grade	2014 (new) source guidelines	Adapte level	2014 (new) recommendation statement
PARM suggests the use of cold therapy in the treatment of sub-acute non-specific low back pain	There is Insufficient Evidence	CLIP^a^ TOP^b^	There is insufficient evidence	ICSI^c^	I	PARM suggests the use of cold therapy in the treatment of sub-acute non-specific low back pain
PARM suggests that patients undergoing active rehabilitation should be provided with as much therapy as possible; a minimum of 1 hour active practice per day, at least five days a week for both physical and occupation therapy	There is Insufficient evidence	NSF^d^	There is evidence	NICE^e^ NZGG^f^ AHA Stroke and TIA^g^	II	PARM endorses that rehabilitation should be given for a minimum of 45 min of active practice per day, 5 days a week, for both physical therapy and occupational therapy. However, the duration and intensity of the program should be adjusted based on the patient’s needs and their ability to participate in an exercise program

^a^TOP: Guideline for the evidence-informed primary care management of low back pain. Edmonton (AB): Toward Optimized Practice

URL: ^b^CLIP: Agency for Health & Social Services. Montreal, Canada: Clinic on Low-Back Pain in Interdisciplinary Practice Guidelines

^c^Adult low back pain. Bloomington (MN): Institute for Clinical Systems Improvement

^d^NSF: National Stroke Foundation. Clinical Guidelines for Stroke Management 2010. Melbourne Australia

URL: ^e^NICE 2013: [32]

^f^NZGG 2010: [33]

^g^AHA Stroke and TIA 2014: Kernan et al. [35]

## Discussion

As far as we are aware, the PARM updating tool is the first of its kind, developed to reinforce the updating process of contextualized clinical guidelines in a developing country. The use of the Johnston et al. [10] tool was reported by Grimmer-Somers and Worley (2010) in updating the Australian and New Zealand Acute Pain Management guidelines [38]. This updating framework fitted well with the PARM requirements, as it could be readily understood by the working group members, and applied standardly to any recommendation and its old and new evidence bases.

Vernooij et al. [8] systematically reviewed 35 guideline manuals and found no consistent or clear information about how to update a guideline. Interestingly this paper did not include the Johnson criteria, which are the clearest step by step approach to date. In a systematic review by Martinez Garcia et al. [6] on the strategies for monitoring and updating clinical practice guidelines, there were four studies which considered the updating process [10–13]. All authors agreed that guideline updating processes were neither time nor resource saving. Eccles [11] updated the CPG by exhaustive research, and classified recommendations as follows: *new* if fresh evidence was identified, *refined* if supplementary evidence was identified, and *unchanged* if now evidence was identified. Parmelli et al. [13] updated recommendations using the GRADE framework, where recommendations were classified as *strong positive*, *weak positive*, *weak negative* and *strong negative* as voted on by the multidisciplinary panel in a series of meetings. A fifth recommendation, ‘no recommendation,’ was eliminated from the categories of strength of evidence. This forced the panelists to take a position on a recommendation even in the absence of strong evidence. Johnston [10] revised recommendations based on the consistency and strength of the evidence. The options reflected the implications of the new evidence on the clinical recommendations. We agreed that this updating process was the most appropriate model in the revision of our contextualized clinical practice guideline.

The PARM updating tool provides a simple, novel and standard approach that considers new evidence’s hierarchy and quality, and revisions of recommendation wordings. It could be used efficiently by other guideline updaters particularly in developing countries, where resources for guideline development and updates are limited. When many people are involved in guideline writing, there is always the possibility of ‘slippage’ in use of wording and interpretation of evidence. The PARM updating tool provides a mechanism for maintaining a standard process for guideline updating processes that can be followed by clinicians with basic training in evidence-based practice principles. The provision of including only high quality reference guidelines in both developing guidelines through contextualization, and updating contextualized guidelines, is one of the strengths of our methodology. A survey by Alonso-Coello [7] showed that the process of updating guidelines is generally not standardized and needs to be more rigorous even if most institutions involved in guideline development have a process for their updates. Our updating approach dovetails with the PARM writing guide [3], which underpinned the development of the foundation contextualized guidelines for stroke and low back pain, rather than a de novo development of a Filipino clinical guideline. We believe that our entire process now offers a resource-efficient process of developing and revising clinical guidelines in order to focus time and resources on evidence dissemination and implementation.

One of the limitations of our approach is that the researchers did not focus on evaluating the currency of the evidence bases of the included CPGs. Shekelle et al. [9] have shown that after 3.5 years, 10 % of the guidelines they reviewed were obsolete, while after 5.8 years, 50 % of the guidelines were outdated. The retrieval of new evidence could either be restricted which would be limited to review, editorials or commentaries of specific journals and expert collaboration [9] or an exhaustive search which was very similar to the process used in the guideline development [10, 39]. In both methods, a methodology group will be needed for skills in systematic search, retrieval and synthesis of evidence which our group did not have. This was largely because of scarcity of resources and time, and voluntary effort from all members. Another limitation of this study would to be potential non-inclusion of high quality guidelines which are not referenced nor published in the internet, as only electronic databases were searched.

## Conclusion

PARM has developed a novel framework to assist the process of revising contextualized clinical practice guidelines, dovetailing this with its initial processes to construct a contextualized CPG. Efficiencies of the PARM updating approach included revisiting the patient journey to validate the critical points which required specific recommendations, and a specific writing guide to revise a recommendation when new evidence was available. With this approach, it is envisioned that updating contextualized CPGs processes in the future will be guided by a simple standardized process, and will be effective and efficient in terms of time, finances and manpower.

## Additional file

10.1186/s13104-015-1588-8 Appendix 1: Consort diagrams for the CPG inclusion process for stroke and low back pain. **Appendix 2:** Sample of how the patient journey was modified between 2012 and 2014

## Abbreviations

AHA: American Stroke Association
APTA: American Physical Therapy Association
CLIP: Clinic on Low-back pain in Interdisciplinary Practice
CPG: clinical practice guideline
GHC: Group Health Cooperative
iCAHE: International Centre for Allied Health Evidence
ICSI: Institute of Clinical Systems Improvement
ISPRM: International Society of Physical and Rehabilitation Medicine
KNGF: Koninklijk Nederlands Genootschap voor Fysiotherapie (Royal Dutch Society for Physical Therapy)
LBP: low back pain
NICE: National Institute for Health and Care Excellence
NZGG: New Zealand Guidelines Group
PARM: Philippine Academy of Rehabilitation Medicine
PHIC: Philippine Health Insurance Corporation
RCC: Royal College of Chiropractors
SASS: South African Stroke Society
TOP: Toward Optimized Practice
