# Supplementary material for: Updating contextualized clinical practice guidelines on stroke rehabilitation and low back pain management using a novel assessment framework that standardizes decisions
Source: BMC Res Notes. 2015 Nov 4;8:643. doi: 10.1186/s13104-015-1588-8 (PMC4632672; doi:10.1186/s13104-015-1588-8)
Supplement: Supplementary file 1 — 10.1186/s13104-015-1588-8 Appendix 1: Consort diagrams for the CPG inclusion process for stroke and low back pain. Appendix 2: Sample of how the patient journey was modified between 2012 and 2014 [file 13104_2015_1588_MOESM1_ESM.docx]

**Appendix 1. FLOW OF LOW BACK PAIN CLINICAL PRACTICE GUIDELINES THROUGH SELECTION PROCESS**

Clinical Practice Guidelines identified using key words: *Clinical Guidelines, Practice Guidelines, low back pain, acute/sub-acute/chronic low back pain, rehabilitation*

N = 11

Did not meet inclusion criteria

N = 4

Not readily available on the internet= 2

Not published in English = 1

Not de novo CPG = 1

Not de novo

Potentially-relevant LBP CPGs

meeting inclusion criteria

N = 7

CPG Excluded due to ICAHE Score <10

N = 3

LBP CPGs with ICAHE score > 10

Relevant for inclusion

N = 4

**FLOW OF STROKE REHABILITATION CLINICAL PRACTICE GUIDELINES THROUGH SELECTION PROCESS**

Clinical Practice Guidelines identified using key words: *Clinical Guidelines, Practice Guidelines, Stroke rehabilitation*

N = 10

Did not meet inclusion criteria

N = 4

Rehabilitation not part of the guideline =3

Did not rank the quality of evidence=1

Not de novo

Potentially-relevant stroke rehabilitation CPGs meeting inclusion criteria

N = 6

CPG Excluded due to ICAHE Score <10

N = 1

Stroke rehabilitation CPGs with ICAHE score > 10

Relevant for inclusion

N = 5

**Appendix 2:** Changes to the patient Journey for Low Back Pain

**(OLD) 2012 Low Back Pain clinical Practice Guideline**

Consultation with Physician

Referral to other Specialists

Persistence of Back Pain

Discharge Instructions

Resolution of Symptoms

Follow-Up

Non-pharmacologic

Treatment

Diagnostic Tests

Identification of Red Flag Signs

Pharmacologic

History/Physical Examination

- Non-specific LBP

- LBP w/ Radiculopathy

-LBP due to other causes

* May be Acute, Sub-acute, or Chronic

**(NEW) 2014 Low Back Pain Patient’s Journey**

Diagnosis

Refer to other Specialists

Follow-Up

Persistence of Symptoms

Resolution of Symptoms

Discharge Instructions

Treatment

- Conservative

- Invasive

Diagnostic Tests

Identify Red Flag Signs

Identify Yellow Flag Signs

History and Physical Examination

Consultation with Physician

- Non-specific LBP

-LBP w/ Radiculopathy

-LBP due to other causes

* May be Acute, Sub-acute, or Chronic
